# PCF11, a Novel CD44-Downstream Transcriptional Target, Linking Its 3’-End Polyadenylation Function to Tumor Cell Metastasis

**DOI:** 10.3389/fonc.2022.878034

**Published:** 2022-06-08

**Authors:** Maryam Al-Mansoob, Salma M. S. Ahmad, Allal Ouhtit

**Affiliations:** Biological Sciences Program, Department of Biological & Environmental Sciences, College of Arts and Science, Qatar University, Doha, Qatar

**Keywords:** PCF11, breast cancer, CD44, hyaluronan, metastasis

## Abstract

Breast Cancer (BC) is the most common and the major health issue in women worldwide. Metastasis, a multistep process, is the worst aspect of cancer and tumor cell invasion is the defining step. Tumor cell invasion requires cell adhesion molecules (CAMs), and alterations in CAMs is considered as an initiating event in metastasis. Among CAMs, CD44 is a large family of more than 100 isoform, and its precise function was initially controversial in BC. Therefore, we have previously established a (Tet)-off inducible expression system of CD44 in MCF-7 primary BC cell line, and showed that CD44 promoted BC invasion/metastasis both *in vitro* and *in vivo*. A microarray gene expression profiling revealed more than 200 CD44-downstream potential transcriptional target genes, mediating its role in BC cell invasion and metastasis. Among these CD44-target genes, the Pre-mRNA cleavage complex 2 protein (PCF11) was upregulated upon the activation of CD44 by its major ligand hyaluronan (HA); This prompted us to hypothesize PCF11 as a potential novel transcriptional target of CD44-promoted BC cell invasion and metastasis. A large body of evidence from the literature supports our hypothesis that CD44 might regulate PCF11 *via* MAPK/ERK pathway. This review aims to discuss these findings from the literature that support our hypothesis, and further provide possible mechanisms linking CD44-promoted cell invasion through regulation of its potential target PCF11.

## Background

One of the most common malignancies in women worldwide is breast cancer (BC), a complex family of diseases associated with a molecular heterogeneity (1). BC cells are commonly known to metastasize into other crucial organs (2). The ability of BC cells to leave their primary tumor site and migrate to a new location, where they form a secondary tumor is dependent on three main processes that include cell adhesion molecules (CAM), proteinases, and growth factors for cell proliferation (3). The process of adhesion relies on CAM that function in cell-cell and cell-extracellular interactions (4). CAM is a wide family of proteins that include immunoglobulins, integrins, cadherins and selectins (5).

A member of the CAM family known as CD44 is the primary receptor of hyaluronan (HA), which is commonly involved in cell signaling mediating cell proliferation, invasion and migration (6). We have previously established a tetracycline (Tet)-Off-regulated expression system of CD44 both in vitro (7) and in vivo (8) to further investigate the role and signaling pathways of CD44-promoted BC cell invasion and metastasis. A 12K CHIP Affymetrix microarray analysis was performed to identify the genes regulated by CD44/HA signaling involved in BC cell invasion (9). Microarray analysis revealed a pool of ˜ 200 potential CD44-target genes associated with its signaling in regulating BC cell invasion. Using a combination of molecular, pharmacological and functional approaches, we have validated a number of these targets, and further dismantled their signaling pathway linking CD44 activation to their transcription (7–11).An additional upregulated gene, the pre-mRNA cleavage complex 2 (PCF11) was selected from the CHIP screen for further investigation based on several lines of evidence supporting the hypothesis that PCF11 is a transcriptional target that underpins CD44-promoted BC tumor cell invasion.

In mammalian cells, the cleavage and polyadenylation (CPA) process is a crucial step for the maturation of mRNA, hence a complex of proteins that work together to perform this step. The CPA complex formed of the cleavage stimulating factor (CstF), the cleavage and polyadenylation specificity factor (CPSF) and the cleavage factor II (CFII) (encompassing PCF11 as a subunit) functions in the termination of transcription (12). PCF11 binds another subunit, Clp1, and plays a major role in regulating the length of the expressed genes (13). Due to its function in CPA and termination, it therefore undergoes autoregulation in order to control cell differentiation, cell adhesion and migration (12). PCF11 regulates vertebrate development (12), and differentiation in neuroblastoma (14). It also controls proliferation, migration and invasion of BC cells (15).

In this review, based on evidence from the literature, we discussed the potential signaling pathways that link PCF11, as a downstream target, to CD44-downstream signaling that promote BC cell invasion and metastasis.

## Structure of Pcf11

PCF11 is located within the long arm of chromosome 11 (11q14.1; starting from base pairs 83,157,095 and ending at base pairs 83,187,451). It comprises about 357 kb of DNA that contains 16 exons (16). PCF11 is usually located within the nucleus and participates in several pathways, including processing of mRNA export pathway, mRNA splicing and RNA Polymerase II Transcription Termination (14). The PCF11 protein encompasses 1555 amino acids with several conserved domains in a helix-turn-helix structure. PCF11 binds to C-terminal domain (CTD) of polymerase II via its CTD interaction domain (CID), subsequently stimulating the phosphorylation of serine 2 that signals the termination of transcription (14). The CID of PCF11 is the right-handed super-helix containing eight α-helices, with an additional C-terminal helix and three anti-parallel α-helices repeats (17). Helices 5 and 6 cause a rotation in which helices 7 and 8 are nearly parallel to helices 3 and 4, which are rotated in a left-handed manner with regards to helices 1 and 2 (17). The arrangement of the CID helices allows certain type of distribution of electron density and hydrophobicity that ultimately provide the domain with extra stability (17). Moreover, it contains conserved Clp1 binding domain and two conserved zinc-binding regions that possess high affinity for RNA (18). The PCF11 protein is a scaffold protein that is mostly recruited to bind other proteins such as Clp1, Rna14, Rna15 (19) and WNK1 (20).

## Functions of Pcf11

As a subunit of the CFII, PCF11 contributes to both pre-mRNA 3’ end processing and termination; It couples the termination of transcription to the export machinery of mRNA (21).

### Physiological Functions of PCF11 in Normal Cells

Nuclear pre-mRNA are subjected to post-transcriptional modification such as the addition of a poly(A) tail to the 3’-end of mRNA. This process involves two main steps: i) the 3’-untranslated region which gives the genes variability, and the ii) the elongation of the poly(A) tail (22). Although CFII contributes to the process with other CPA proteins, its subunits play major roles unlike other proteins. Specifically, PCF11 is one of the scaffolding proteins in the complex and the only subunit able to interact with all other three subunits, including Rna14, Rna15 and Clp1 (23). Moreover, PCF11 interacts directly with the subunits of cleavage and polyadenylation factor (CPF), and the CTD of Pol II, which couples the transcription elongation complex to the processing machinery (24). The aforementioned interactions occur via the N-terminal CID of PCF11, which interacts with the CTD of Pol II, the C-terminal Clp1 interaction domain and the central domain allowing its interaction with Rna14-Rna15 as a homodimer (24)The CID-CTD interaction between PCF11 and RNA polymerase is what dismantles elongation complexes in vitro, and promotes polymerase’s CTD serine-2 phosphorylation in yeast (12). Furthermore, depletion of PCF11 alone abolished the early termination activity of the complex and allowed transcriptional readthrough past the end sites and extended the 3’ end, proving the role of PCF11 in proximal APA shifts (12).

remarkably, it has been reported that the poorly invasive MCF7 cells express short genes, while highly metastatic MDA-MB-231 express long genes; the major function of PCF11 in 3’UTR cleavage and polyadenylation, when inhibited resulted in the production of long genes via intronic polyadenylation. All these findings put together indicate that PCF11 3’UTR processing function is associated with the highly metastatic phenotype (25).

**Figure 1:**
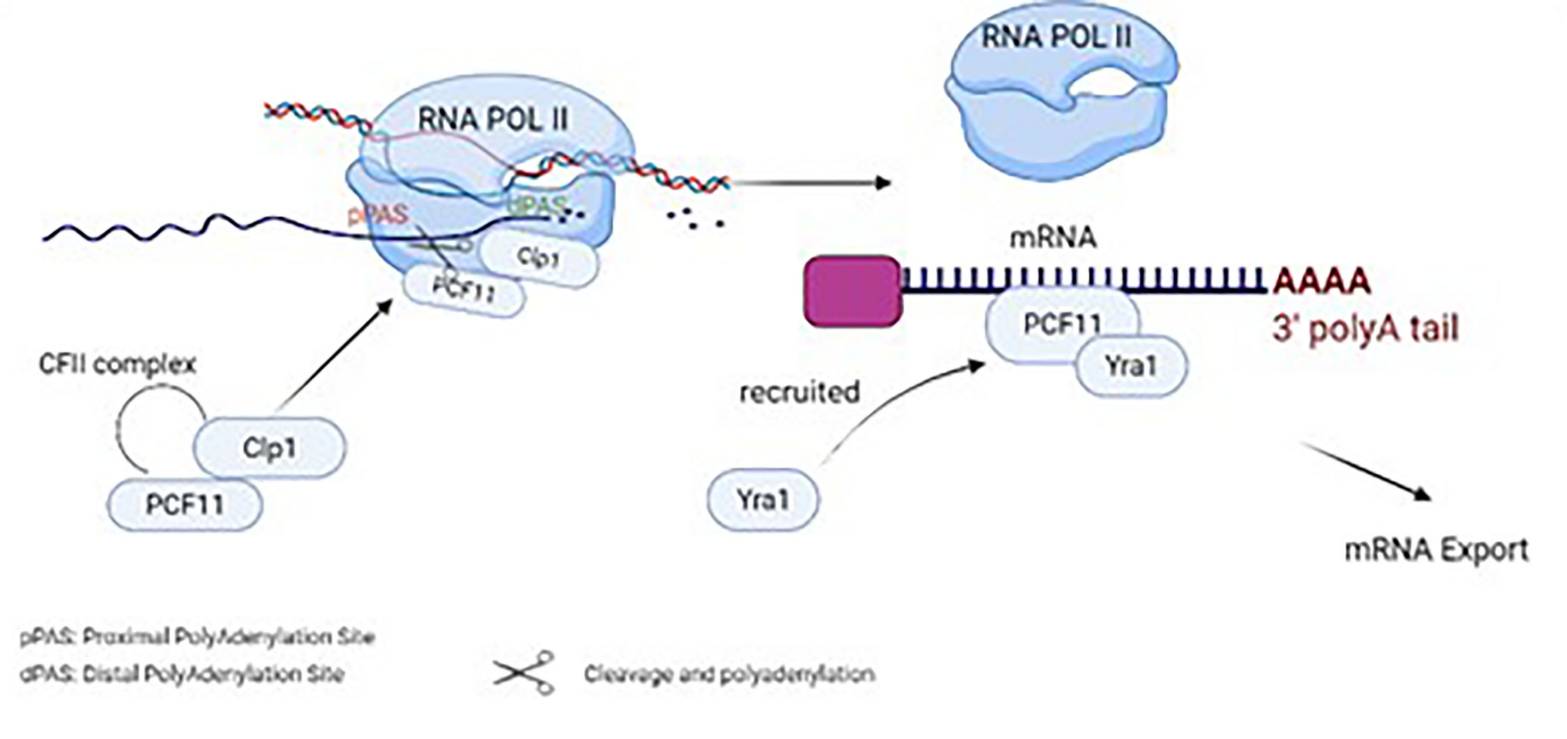
Physiological functions of PCF11 in normal cells and its role in cleavage, polyadenylation, termination and mRNA export..

Moreover, PCF11 can couple mRNA export with the termination of transcription (26). The mRNA export machinery is associated with RNA and RNA Export factors (REF) such as Yra1, commonly recruited by an ATPase/RNA helicase known as Sub2. Yra1 then works in pairs with the RNA binding protein Aly, which delivers the mRNA to the export receptors. Yeast models proved physical linking of Yra1 recruitment to PCF11, through direct protein-protein interaction with the zinc finger/Clp1 region of PCF11 (26). Additionally, biochemical assays suggest overlapping regions between PCF11 and the export receptor Mex67 and Sub2, indicating its role in coupling the mRNA export to transcription termination (26). Additionally, biochemical assays suggest overlapping regions between PCF11 and the export receptor Mex67 and Sub2, indicating its role in coupling the mRNA export to transcription termination as shown in (**Figure 1**).

### Functions of PCF11 in Cancer and Its Association to CD44

Although the functional role of PCF11 in BC is poorly understood and the underlying mechanisms remain nascent (27), it promotes invasion and migration in a triple-negative mouse BC cell line known as 4T1 (15). In fact, 4T1 cell line is a metastatic cell with high migration and invasion abilities; however, knockdown of PCF11 in 4T1 reduced their migration and invasion by 70% (15). In MCF7 cell lines treated with the anti-cancer cannabidiol, PCF11 was amongst the significantly downregulated proteins ( (27). More interestingly, CD44 activates WNK1-induced BC cell migration through its involvement in MAPK/ERK pathway, as its knockdown suppressed ERK (28). On the other hand, PCF11 has a WNK1 binding domain (20) and CD44 regulates the MAPK/ERK pathway (29). Thus, these data support our hypothesis that CD44 might regulate PCF11 via MAPK/ERK pathway.

**Figure 2:**
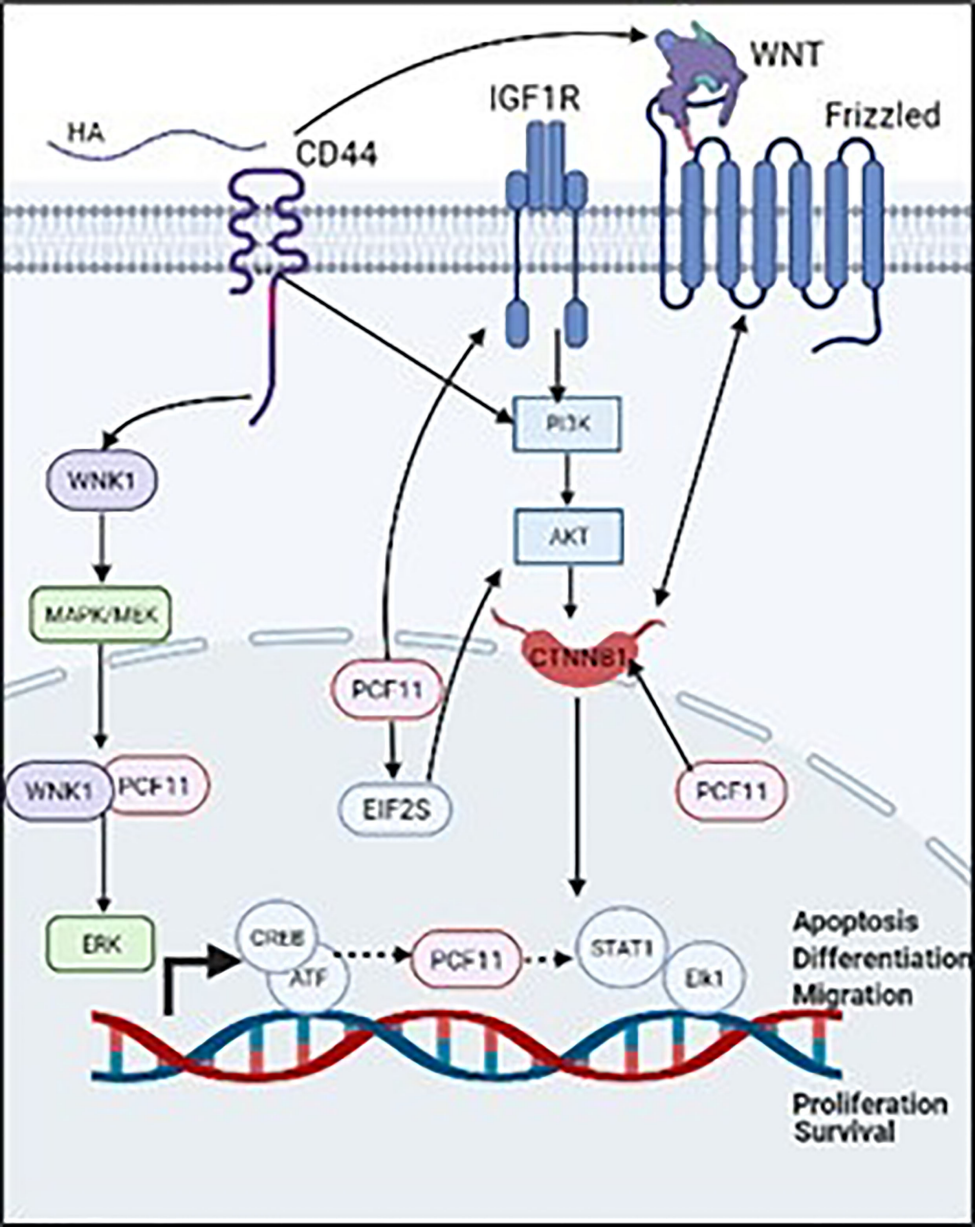
Validated (→) and proposed (⤑) mechanisms that associate PCF11 to CD44/HA signaling promoted BC cell invasion.

Alternative polyadenylation (APA) factors can regulate various cancers (30). Amongst these APA factors, PCF11 is involved in several cancers as in **Table 1**, including neuroblastoma (25) liver, lung and BC (33). In mouse embryo fibroblasts, knockdown of PCF11 reduced proliferative genes (15). Also, PCF11 is suggested as a marker in prostate cancer, in which treatment with the prostate cancer preventive agent genistein downregulates PCF11 in LNCaP cell lines (32). In neuroblastoma, high levels of PCF11 regulate differentiation, proliferation, apoptosis and cell cycle, while low levels of PCF11 are associated with favorable outcomes and spontaneous tumor regression (34). Depletion of PCF11 in human neuroblastoma cell lines, abolished colony formation, induced retarded tumor growth and reduced invasiveness (31). Studies have reported that PCF11 induces invasiveness in neuroblastoma through mediating WNT signaling via beta-catenin 1 (CTNNB1), subsequently activating PI3K/AKT that regulates, cell cycle progression, proliferation and apoptosis (35). In addition, knockdown of PCF11 significantly inhibited WNT signaling (31). High levels of PCF11 significantly upregulate EIF2S1 and IGF1R (31). IGF1R is an insulin growth receptor that directly interacts with PI3K/AKT to induce malignant phenotypes in neuroblastoma (35). Furthermore, bioinformatics tools revealed several transcriptional factors in association with PCF11 including transcriptional factors induced by MAPK/ERK and PI3K/AKT signaling pathways such as, STAT1, Elk1 and CREB/ATF, respectively (36). Curiously, CD44 promotes breast tumor cell invasion and migration via regulation of the WNT signaling pathway (37). It also promotes cytoskeletal remodeling, survival, growth and invasion via activation of the PI3K/AKT pathway (8, 10) as shown in (**Figure 2**).

**Table 1:** Summary of the role of PCF11 in different cancers.

Cell line	Cancer/cell type	Role of PCF11	References
MCF7	Human epithelial breast cancer cells	MCF7-treated with Cannabidiol an anti-cancer agent significantly downregulated PCF11.	(27)
4T1	Mouse triple negative Breast cancer cells	Knockdown of PCF11 caused 70% reduction in migration and invasion.	(15)
NIH 3T3	Mouse embryo fibroblasts	Knockdown of PCF11 reduced cell proliferation.	(15)
CHP-134 & BE(2)-C	Human neuroblastoma cells	PCF11 depletion suppressed tumor growth, abolished colony formation and reduced invasiveness	(31)
LNCaP	Human prostate adenocarcinoma cells	Genistein, a prostate cancer preventive agent, downregulated PCF11.	(32)

## Conclusion

A large body of evidence from the literature supports our hypothesis that PCF11 might be a novel transcriptional target that underpins CD44-downstream signaling promoting BC cell invasion/metastasis. As discussed above, PCF11 promote cancer progression via regulation of the mechanisms controlling cell proliferation, migration, and invasion. While in BC, PCF11 is part of the MAPK/ERK pathway (28), it is part of the WNT/PI3K/AKT signaling pathway in neuroblastoma (31). On the other hand, CD44 was validated as a key regulator of WNT (37), PI3K/AKT (8) and MAPK/ERK signaling pathways (29), thereby supporting our hypothesis that PCF11 is a potential novel transcriptional target that underpins CD44/HA-promoted tumor cell invasion. Ongoing in vitro experiments in our laboratory, aim to identify and validate the molecular players that link the activation of CD44, by its ligand HA, to the transcriptional regulation of PCF11 3’UTR to promote tumor cell invasion and metastasis.

## Author Contributions

MA-M: Writing‐original draft (lead). SMSA: Editing. AO: Conceptualization (lead); Funding acquisition (lead); Writing‐review & editing. All authors contributed to the article and approved the submitted version.

## Funding

Qatar University Internal grant number funded this research: QUST-1-CAS2019-22, QUST-2-CAS-2022-486 QUST-2-CAS-2022-487 Qatar Foundation grant number: UREP24-117-1-027 and APC. The Qatar National Library provided open Access funding.

## Conflict of Interest

The authors declare that the research was conducted in the absence of any commercial or financial relationships that could be construed as a potential conflict of interest.

## Publisher’s Note

All claims expressed in this article are solely those of the authors and do not necessarily represent those of their affiliated organizations, or those of the publisher, the editors and the reviewers. Any product that may be evaluated in this article, or claim that may be made by its manufacturer, is not guaranteed or endorsed by the publisher.

## Abbreviations

AKT’, Protein kinase B; BC, Breast cancer; CAM, Cell adhesion molecule; CD44, Cluster of differentiation 44; HA, Hyaluronan; F11, Pre-mRNA cleavage complex 2 protein; PI3K, phosphoinositide 3-kinase; Ras, Rat sarcoma; Tet, Tetracycline; CPA, cleavage and polyadenylation; CstF, cleavage stimulating factor; CPSF, cleavage and polyadenylation specificity factor; CF II, cleavage factor II; CTD, C-terminal domain; CID, CTD interaction domain; CTNNB1, beta-catenin; Clp1, cleavage factor polyribonucleotide kinase subunit 1; APA, Alternative polyadenylation; REF, RNA and Export factor; WNK1, WNK lysine deficient protein kinase 1; MAPK, Mitogen-activated protein kinase; ERK, extracellular-signal-regulated kinase.
